# The Effect of Diabetes Mellitus on Short Term Mortality and Morbidity after Isolated Coronary Artery Bypass Grafting Surgery

**Published:** 2013-06-01

**Authors:** Vahideh Koochemeshki, Hamid Reza Salmanzadeh, Hojjat Sayyadi, Morteza Amestejani, Shahyad Salehi Ardabili

**Affiliations:** 1 Department of Cardio Surgery, Seyed Al-shohada Cardiovascular Center, Urmia University of Medical Sciences, Urmia, IR Iran; 2 Department of Biostatistics, Shahid Beheshti University of Medical Sciences, Tehran, IR Iran

**Keywords:** Diabetes, Postoperative Complications, Coronary Artery Bypass Graft, Morbidity, Mortality

## Abstract

**Background:**

This study was conducted to determine whether Diabetes Mellitus (DM) is a predictor of short term mortality ; morbidity, or early readmission to hospital after Coronary Artery Bypass Graft (CABG).

**Methods:**

We analyzed a large cohort of 952 patients who had undergone isolated CABG. The preoperative, intera operative and postoperative risk factors as well as the complications and 30-day mortality rates were compared between the diabetics and non-diabetics. Among the 952 patients; 734 ones (77.1%) were in non-diabetic group and 218 (22.9%) were diabetics.

**Results:**

Having DM did not increase the risk of 30-day mortality. In addition, DM did not affect the major complications; arrhythmia, Myocardial Infarction(MI), infective complications, neurological complications, Pulmonary Embolism (PE) except renal complications that was higher in the diabetics (5.5% vs 1.4%; P<0.001, OR=4.2) However reoperation for bleeding was higher in non-diabetic patients (7.9% vs 4.6%; P=0.009, OR=1.7). Nevertheless ,no significant difference was observed between the two groups regarding mechanical ventilation time (hour), reintubation, length of ICU stay (day), length of hospital stay (day), and readmitting as postoperative variables.

**Conclusions:**

Except for renal complications, DM was not associated with adverse outcomes in the patients undergoing isolated CABG.

## 1. Background

Diabetes Mellitus (DM) is an established risk factor for the development of Coronary Artery Disease (CAD) and approximately 20% to 30% of the patients undergoing Coronary Artery Bypass Graft (CABG) have DM (1). There is not enough evidence to determines the impact of DM on short-term mortality and morbidity in the patients undergoing CABG. One study (1) suggested that in-hospital mortality was elevated in DM, although the results were inconsistent (2-13). The patients with diabetes have more advanced; diffuse coronary artery stenosis and more end-organ dysfunction, including renal insufficiency and neurologic deficits (14). Clinicians who treat the diabetic patients with CAD should be aware of the effects this condition has on the outcomes of CABG surgery (15). This study is designed to determine the influence of DM on post operative mortality, morbidity, length of hospital stay, and readmission after isolated CABG.

## 2. Materials and Methods

The present historical cohort study aimed to the collect and analyzes data of 952 consecutive patients who underwent isolated CABG procedure at Seyed Al-shohada Cardiovascular Center and Imam Khomeini hospital in Urmia between July 2004 and November 2011.

The patients with a documented history of diabetes regardless of types who needed anti-diabetic agents either on admission or preoperative diagnosis were classified as having DM.

Among the 952 patients, 734 ones (77.1%) were in the non-diabetic group and 218 (22.9%) were diabetic. The preoperative, operative, and postoperative variables were prospectively collected and listed in Tables 1, 2, and 3.

**Table 1.** Preoperative Patient’s Variables

Variable	Diabetes Patients (n=218)	Non-Diabetes (n=734)	*P *value
Age (mean ±SD)	58.6±9	59.3±9	0.914
Sex (m/f) (%)	53/47	75/25	<0.001
Hemoglobin	13.5±7	13.5±1.5	0.684
LVEF[Table-fn fn5096](mean ±SD)	41.5±11	41.3±10	0.618
Hypertension (%)	51.4	38.4	<0.001
Smoking (%)	15.6	18.3	0.074
COPD[Table-fn fn5096](%)	8.3	7.1	0.095
Renal insufficiency (%)	0.9	1.6	0.068
Mean of Body Mass Index	27.7	26.3	0.045

Abbreviations: LVEF, Left Ventricular Ejection Fraction; COPD, Chronic Obstructive Pulmonary Disease

**Table 2.** Operative Patients’ Variables

Variable	Diabetes Patients (n=218)	Non-Diabetes (n=734)	*P *value
Operative time (min) (mean±SD)	434±81	422±76	0.014
Aortic cross clamp time (min) (mean±SD)	82±25	83±30	0.351
No. of coronary grafts (%)			0.143
One	3.7	4.1	
Two	16.5	19.6	
Three	79.8	75.5	
≥Four	0	0.8	

**Table 3.** Postoperative Patients’ Variable

Variable	Diabetes Patients (n=218)	Non-Diabetes (n=734)	*P* value
Emergency case (%)	1.8	2.5	0.096
Reintubation (%)	95.4	97.5	0.068
Arrhythmia (%)	13.8	14.4	0.229
Myocardial infarction (%)	0	1.1	0.072
Reoperation for Bleeding (%)	4.6	7.9	0.009
Renal complications (%)	5.5	1.4	<0.001
Neurological Complications (%)	O.9	0.3	0.059
Infective Complications (%)	4.6	4.4	0.091
Length of ICU stay (mean ±SD)	3.8±1.9	3.7±1.7	0.692
Length of Hospital stay (mean ±SD)	11.3±5.5	10.3±8.3	0.073
Short tem death (%)	4.6	2.7	0.061
PE[Table-fn fn5099](%)	5.5	4.1	0.102
Mechanical ventilation time (hr)	6±6	7±9	0.117
Readmitting (%)	8.3	6	0.161

Abbreviations: PE, Pulmonary Embolish

BMI was defined as the weight in kilograms divided by the height in meters squared. Smokers were defined as having used more than 15 cigarettes per day for more than 5 years. Chronic Obstructive Pulmonary Disease (COPD) was indicated if the patient was treated with the medications for COPD. In addition, preoperative renal failure was specified as serum Creatinin >2 mg/dL.Besides arrhythmia was considered if associated with medication prescribed for hemodynamic compromise. Myocardial Infarction (MI) was documented by a rise in cardiac isoenzyme values or electrocardiographic changes indicative of MI and confirmed by a physician. Postoperative bleeding was implied if the patient required re-exploration to assess bleeding. Further more, renal complications included a rise in Creatinin concentration of 1 mg/dL above the baseline value or the continued presence of preoperative renal failure. Postoperative neurological complications were recorded by a neurologic consultant and included both transient ischemia attacks and strokes. Infective complications included septicemia and deep sternal and leg wound infection as defined by positive culture and requiring antibiotic therapy (15). Finally, operative mortality was shown as any death occurring during the hospital stay or within 30 days after the surgery.

Statistical analysis was performed using the SPSS statistical software (version19). The study groups were compared using Student’s t-test for the continuous variables and the χ^2 ^test or Fisher exact test for the categorical variables. The continuous variables were presented as mean±standard deviation. In all the cases, *P* value<0.05 was considered statistically significant. Binary logistic regression test was used to determine the significant predictive factors for short tem death and postoperative length of hospital stay>10 days similar to other studies conducted on the issue (5, 7, 9). In table 4, short time death is the dependent variable and which is a qualitative (binary) one. Also, the covariate variables which predict short tem death were sex, reoperation, infective complications, length of ICU stay, and age. Our base lines in Table 4 were male gender for sex, none reoperation, and none infective complication. In table 5, length of hospital stay>10 days is the dependent variable which is a qualitative (binary) one. Also, the covariate variables predicting it were sex, smoking, renal complications, operation time, reoperation, readmitting, and reintubation. Our base lines in Table 5 were male gender for sex, none smoker, none renal complications, none reoperation, none reintubation; and none readmitting.

## 3. Results

Table 1 compared the data for preoperative patient variables. The two study groups had no significant incidence of COPD, Renal Failure (RF), Left Ventricular Ejection Fraction (LVEF), and Hemoglobin (Hb). The data showed that the prevalence of diabetes was lower in males compared to females 36.6% of females but only 17.3% of males were diabetic (*P*<0.001). Also a significant difference was found between the two groups regarding hypertension and BMI that both of them were more in diabetics.

Considering the operative factors in Table 2, no significant differences were found between the diabetics and non-diabetics regarding the rates of aortic cross clamp (min) , and the number of coronary grafts (%). However a significant difference was observed between the two regarding the operation time; the diabetics had a higher operation time compared to other group (434±81vs 422±76; P =0.014).

It should be noted that no significant difference was found between the two groups concerning the findings of the present study revealed no significant difference between the two groups regarding mechanical ventilation time (hour), reintubation, arrhythmia, neurological complications, PE, readmitting, myocardial infraction (MI), infective complications as postoperative variables. Nonetheless, renal complications were significantly higher in the diabetic patients compared to the non-diabetic ones (5.5% vs 1.4%; *P*≤0.001 , OR=4.2).

It should be noted that no significant difference was found between the two groups concerning the length of ICU stay (day) and length of hospital stay (day). Table 3 showed that the non-diabetic patients had more reoperations for bleeding (7.9% vs 4.6% ; P=0.009 , OR=1.7). Also, no significant difference was observed between the study groups regarding short term mortality rate.

The result of binary logistic regression analysis showed that female gender, reoperation, infective complications, and increasing length of ICU stay, and rising age predicted short tem death (Table 4) while chance death for female gender was 2.2 times to males. Chance death for the patients with reoperation and infective complications were respectively 8.2 and 4.9 times more than others respectively. Moreover the chance death was raised fo6% per of age and was raised 40% per day in ICU.

**Table 4.** Multivariable Analysis of the Predictors of 30-day Death

Variable	Coefficient Regression	*P* value	OR	95% CI*
Sex	0.79	0.046	2.2	1.8-5.63
Reoperation	2.11	<0.001	8.2	3.29-20.7
Infective Complications	1.59	0.023	4.9	1.25-19.25
Length of Icu stay	0.44	<0.001	1.4	1.32-1.82
Age	0.58	0.043	1.06	1.002-1.12

^*^95% confidence interval for OR

The result of binary logistic regression analysis showed that female gender, smoking, renal complications, reoperation, readmitting, reintubation, and rising operation time predicted the postoperative length of hospital stay>10 days (Table 5) while the chance risk for female gender was 2.43 times more than that males. The chance risk for the patients with renal complications, readmitting and reintubation were respectively 3.96, 2.58, and 2.59 times more than others. Also, reoperation and smoking caused 99%and 56% increase in the chance risk respectively.

**Table 5.** Multi variable Analysis of the Predictors of Postoperative Length of Hospital Stay>10 days

Variable	Coefficient Regression	Odds ratio	95% CI*	*P* value
Sex	0.88	2.43	1.72-3.42	<0.001
Smoking	0.44	1.56	1.01-2.38	0.02
Renal complications	1.37	3.96	1.04-15.05	0.043
Operation time	0.09	1.009	1.007-1.011	<0.001
Reoperation	0.68	1.99	1.05-3.77	0.035
Readmitting	0.94	2.58	1.41-4.69	0.002
Reintubation	0.95	2.59	0.84-7.98	0.049

^*^95% confidence interval for OR

## 4. Discussion

The World Health Organization has estimated that the global burden of diabetes would increase from 135 million in 1995 to 299 million patients by the year 2025 (16). DM is a major risk factor for cardiovascular disease, and arteriosclerosis is responsible for 80% of short term deaths in the patients with DM, Also approximately 20% to 30% of the patients undergoing CABG have DM (17). Some studies have assumed diabetes as a risk factor for a poor outcome of operative mortality following CABG (9,17,18). In some other studies similar to our findings, no significant relationship was found between diabetes and mortality (2-8,10-13,19,20).Our results and those of Moshtaghi’s study (20) did not support the research by Carson et al. (17) which showed DM increased morbidity following CABG.

Moshtaghi and Carson (17,18,20) concluded that the diabetic patients were more likely female and, obese, and had hypertension which is in line with our findings. However, in contrast to those studies, the diabetic patients were not older and did not have lower LVEF and renal failure in the present study. We found that the prevalence of COPD was similar in the two groups which is in agreement with other studies (17). Also, there were a lower number of smokers among our diabetic patients which is consistent with the findings of some researches (17,20).

However, our study findings differed from the report by some researchers (20-22)who found that mechanical ventilation time in the diabetics was more than the non-diabetics.

Carson and Kubal (17,18) suggested that the diabetic patients had a higher length of hospital stay ,but we did not find any association between the two which is similar to the results of the study by Ngaage et al. (23). The present study also showed no significant increase in the incidence of atrial arrhythmia which is in agreement with another research performed in IR Iran (20) .Also, we found no association between the incidence of MI and diabetes in our patients while according to colon et al. (24), the incidence of MI in diabetics was lower than the other group. The findings of the current study revealed a significant association between diabetes and renal complication which has been supported by some other studies, as well (18,24,25). It is also interesting to consider the possibility that the metabolic abnormalities associated with DM are responsible for some of the increased mortalities and morbidities. Dehydration and electrolyte disturbances resulting from uncontrolled hyperglycemia may also cause renal complications (20).

 Our study demonstrated no significant difference between the two groups regarding the neurological complications which is similar to the findings of the study by Kubal et al. finding (18) .However, some studies have shown the neurological complications to be higher in diabetics (6,17, 20,24).Interestingly, the diabetic group compared to the non-diabetics was significantly less likely to experience postoperative bleeding; of course this result was not confirmed by Moshtaghi et al. (20).

 Many prior studies (9,17,26) have shown that the diabetic patients had a higher incidence of infective complications. Decreased perfusion of subcutaneous fat tissue, postoperative hemorrhage, prolonged operation time, age, renal failure, and low cardiac output syndrome may increase the infective complications (15).However, we found a different result that infective complications was not more in the diabetic group which is similar to the findings of the study by Rajakaruna et al. (27). In general, treatment of hyperglycemia in the postoperative period might reduce infection in the patients with DM (15). In contrast to the previous studies the study, risk factors for infection including age, renal failure, COPD, post operative hemorrhage, and low cardiac output (15) were not high in the diabetic group in this study (28,29). Thus, significant association was found between diabetes and infection, which was attributed to the greater extent of these factors in the diabetic group.

## 5. Conclusion

The findings of the present study showed no association between DM and a lot of postoperative outcomes, but DM had a significant impact on renal complication. In this study, no difference was observed between the diabetics and non-diabetics regarding short term mortality rate. Long term follow up is needed to find the late complications in the diabetic patients after CABG.
